# Zn- and Ti-Doped SnO_2_ for Enhanced Electroreduction of Carbon Dioxide

**DOI:** 10.3390/ma14092354

**Published:** 2021-05-01

**Authors:** Katarzyna Bejtka, Nicolò B. D. Monti, Adriano Sacco, Micaela Castellino, Samuele Porro, M. Amin Farkhondehfal, Juqin Zeng, Candido F. Pirri, Angelica Chiodoni

**Affiliations:** 1 Center for Sustainable Future Technologies @POLITO, Istituto Italiano di Tecnologia, Via Livorno 60, 10144 Turin, Italy; nicolo.monti@iit.it (N.B.D.M.); adriano.sacco@iit.it (A.S.); Amin.Farkhondehfal@iit.it (M.A.F.); juqin.zeng@iit.it (J.Z.); fabrizio.pirri@iit.it (C.F.P.); angelica.chiodoni@iit.it (A.C.); 2 Department of Applied Science and Technology, Politecnico di Torino, C.so Duca degli Abruzzi 24, 10129 Turin, Italy; micaela.castellino@polito.it (M.C.); samuele.porro@polito.it (S.P.)

**Keywords:** electrochemical CO_2_ reduction, doped SnO_2_ catalyst, mesoporous, oxygen vacancy, HCOOH production

## Abstract

The electrocatalytic reduction of CO_2_ into useful fuels, exploiting rationally designed, inexpensive, active, and selective catalysts, produced through easy, quick, and scalable routes, represents a promising approach to face today’s climate challenges and energy crisis. This work presents a facile strategy for the preparation of doped SnO_2_ as an efficient electrocatalyst for the CO_2_ reduction reaction to formic acid and carbon monoxide. Zn or Ti doping was introduced into a mesoporous SnO_2_ matrix via wet impregnation and atomic layer deposition. It was found that doping of SnO_2_ generates an increased amount of oxygen vacancies, which are believed to contribute to the CO_2_ conversion efficiency, and among others, Zn wet impregnation resulted the most efficient process, as confirmed by X-ray photoelectron spectroscopy analysis. Electrochemical characterization and active surface area evaluation show an increase of availability of surface active sites. In particular, the introduction of Zn elemental doping results in enhanced performance for formic acid formation, in comparison to un-doped SnO_2_ and other doped SnO_2_ catalysts. At −0.99 V versus reversible hydrogen electrode, the total faradaic efficiency for CO_2_ conversion reaches 80%, while the partial current density is 10.3 mA cm^−2^. These represent a 10% and a threefold increases for faradaic efficiency and current density, respectively, with respect to the reference un-doped sample. The enhancement of these characteristics relates to the improved charge transfer and conductivity with respect to bare SnO_2_.

## 1. Introduction

Climate change causes are attributed by scientific community to the increased production of carbon dioxide (CO_2_) by anthropogenic activity. CO_2_ is in fact one of the principal greenhouse gases which are contributing to global warming, and in order to minimize the continuous growth of its atmospheric concentration, it can be used as raw material to obtain products with high energy value. This can be achieved via various processes including electrochemical CO_2_ reduction with the use of proper catalysts.

Among many products that can be obtained (depending on the catalyst characteristics, the reaction conditions and the electrolyte used), the CO_2_ reduction reaction (CO_2_RR) to carbon monoxide (CO) or formic acid (HCOOH) is up to now the economically most viable process that can challenge conventional production routes [1]. In particular, formic acid with its low toxicity, availability, and convenient handling results in a great range of applications in agriculture and chemical industry fields [2]. Moreover, it is considered as one of the most promising materials to be used in the future transport sector as hydrogen carrier [3]. Considering its relatively high industrial price and the increasing market demand, the high potential for formic acid (or formate) production by electrochemical CO_2_RR at low energy consumption becomes extremely interesting [4,5].

Among others, tin oxide-based materials are known to catalyze the electrochemical reduction of CO_2_ to CO and HCOOH. From a research point of view, excellent properties, including high faradaic efficiencies (FEs) and current densities for CO_2_RR, were observed [6,7,8]. However, for practical application there is need to further improve the values of current density, FE and working potential [4]. This could be achieved by various routes, including nanostructuring of the electrocatalysts with tailored surface configuration [9,10,11,12] or doping [13]. The latest approach was proposed by Saravanan et al., who showed through theoretical calculations that doping with several elements, including Ti, V, Zr, Nb, Cd, and Zn, produces upward shifts in the Pourbaix diagram boundary separating the [3H/1CO_2_] and [4H/1CO_2_] states, which in turn result in lowering the overpotential for CO_2_ reduction with respect to un-doped tin oxide [13].

Previous reports on application of doped SnO_2_ to CO_2_ electrocatalysis show that doping SnO_2_ with Mn generates a large amount of oxygen vacancies and results in improved performance [14]. Similarly, the co-doping with Bi and S results in improving FE for CO_2_RR, with lower overpotential and higher partial current density with respect to un-doped SnO_2_ [15]. Other works include the introduction of N into the SnO_2_ lattice [16] and the co-doping with Cu and S [17]. To the best of our knowledge, no reports on the doping of SnO_2_ with Zn and Ti elements for CO_2_RR are available, although it was used for other applications, including optics [18], electronics [18], and widely in gas sensing [19]. Ti-doped SnO_2_ was employed as photocatalyst, demonstrating enhanced activity for degradation of organic dyes, due to reduced recombination of the electron-hole pairs and expanded range of light absorption [20]. Moreover, Wang et al. demonstrated that the surface of Zn-doped SnO_2_ contains oxygen vacancies and favors the oxidation of methanol to carbon oxides, while Ti-doped oxide promotes the yield of dimethyl ether [21]. These articles, together with the theoretical prediction of improved properties after the insertion of Zn and Ti into SnO_2_, abundance of these elements and their relatively low toxicity, suggest that this can be a successful and environment friendly way to enhance the electrocatalytic performance of tin oxide.

In this work, we report a facile synthesis method to fabricate chainlike mesoporous SnO_2_ via anodic oxidation doped with Zn or Ti via wet impregnation and atomic layer deposition (ALD). The composition, morphology, crystalline structure of the doped SnO_2_ nanocatalysts were characterized using scanning electron microscopy (SEM), transmission electron microscopy (TEM), X-ray diffraction (XRD), micro-Raman spectroscopy, and X-ray photoelectron spectroscopy (XPS). Linear sweep voltammetry (LSV), electrochemical impedance spectroscopy (EIS), and chronoamperometric (CA) measurements were conducted to elucidate the catalytic performance of the electrodes for the CO_2_RR, also comparing the results to those of a reference sample prepared using the same synthesis route, without insertion of any dopant. The proposed doping methods may provide a novel strategy to promote the development of electrochemical reduction of CO_2_.

## 2. Materials and Methods

### 2.1. Catalyst Preparation

The catalysts were prepared by anodic oxidation of tin foil (0.5 mm thick, purity 99.95%, Advent RM, Oxford, UK) in 0.3 M NaOH using a voltage of 10 V, under continuous stirring in an ambient environment, employing a platinum foil as cathode. Prior to the synthesis, the foils were ultrasonically cleaned in acetone and in ethanol and dried with a N_2_ flow. At the end of the oxidation process, the anodes were rinsed in distilled water and dried with N_2_. The synthesis procedure is detailed in a previously published work [12].

Two approaches were used for doping of the SnO_2_ catalyst. In the first, the as-obtained samples were soaked in a proper solution, which was continuously stirred for two hours. Two solutions were employed for the doping with Zn and Ti, namely, 1.2 mM zinc acetate (Merck, Darmstadt, Germany) in distillated water [22] and 20.3 mM titanium isopropoxide (Sigma-Aldrich, Munich, Germany) in isopropyl alcohol (Sigma-Aldrich) [23], respectively. In the second approach, Zn doping was obtained using a Beneq tool for atomic layer deposition, model TFS 200 [24,25,26], using extended ALD cycles in order to achieve complete infiltration into the mesoporous SnO_2_ structure. Each ALD cycle was composed by exposure to diethylzinc precursor for 3 s, purging for 15 s, exposure to water co-reactant for 3 s, purging for 20 s. The cycle was repeated 4 times, corresponding to 4 ZnO layers on flat substrate, and the deposition temperature was kept at 100 °C during the whole process.

Such prepared samples were then thermally treated in ambient atmosphere at 370 °C for 2 h with a heating ramp of 150 °C h^−1^, using a Nabertherm LT 15/12/P330 (Nabertherm, Lilienthal, Germany) muffle furnace. This process was performed to improve the intercalation of the doping species [27]. Afterwards, a doped tin oxide powder was produced by sonicating the samples in ethanol until all the synthesized oxide was detached from the metal tin foils. The obtained suspension was then centrifuged several times and then dried overnight. Finally, the catalyst material was further heated at 600 °C for 4 h, which is higher than this in a previously published work [12], in order to promote the interaction of the doping species with tin oxide, and convert it into a tetragonal rutile SnO_2_ crystalline structure [28].

The SnO_2_ catalyst impregnated with Zn and Ti were named “ZnWet” and “TiWet” respectively, while the one processed by ALD was named “ZnALD”. Un-doped SnO_2_ (named “Reference”) was also synthetized employing the same procedure without the doping step.

### 2.2. Electrode Preparation

The electrodes for CO_2_RR were prepared by drop casting on a carbon paper equipped with a gas diffusion layer (GDL; SIGRACET 28BC, SGL Technologies, Meitingen, Germany). The ink was made by 10 mg of the obtained catalyst, 1.5 mg of black carbon (Vulcan XC-72R, Cabot, Boston, MA, USA), 320 µL of isopropyl alcohol (Sigma-Adrich) and 100 µL of Nafion 117 (5 wt %, Adrich). Before deposition, all ingredients were thoroughly mixed and sonicated for 30 min until a uniform slurry was obtained. The final catalyst loading on the electrode is 3.3 mg cm^−2^.

### 2.3. Characterization of Materials

Field-emission scanning electron microscopy (FESEM, ZEISS Auriga, Oberkochen, Germany) was used to evaluate the morphology of the as-prepared material and doped catalysts. Transmission electron microscopy (FEI Tecnai G2 F20 S-TWIN, Thermo Fisher Scientific, Waltham, MA, USA) analysis was performed with a field emission gun, operating at 200 kV. High angle annular dark field (HAADF), and energy dispersive X-ray spectroscopy (EDX, EDAX) detectors were used in scanning TEM (STEM) mode. For TEM characterization, the catalysts were dispersed in ethanol and then deposited on standard holey carbon Cu TEM grids. X-ray diffraction was performed in Bragg−Brentano symmetric geometry by using a PANalytical X’Pert Pro instrument (Malvern Panalytical, Malvern, UK) (Cu Kα radiation, 40 kV and 30 mA) equipped with an X’Celerator detector. Rietveld analysis of the XRD spectra was done using the Materials Analysis Using Diffraction (MAUD) software (version 2.94) [29,30]. The instrumental function was estimated using a standard sample (LaB_6_). X-ray photoelectron spectroscopy was carried out by using a PHI 5000 VersaProbe (Physical Electronics, Chanhassen, MN, USA) system. The X-ray source was a monochromatic Al Kα radiation (1486.6 eV). Spectra were analyzed using Multipak 9.7 software. All core-level peak energies were referenced to C1s peak at 284.5 eV (C−C/C−H) and the background contribution in high-resolution (HR) scans was subtracted by means of a Shirley function. Raman spectroscopy was carried out with inVia Reflex micro-Raman (Renishaw, Gloucestershire, UK) spectrophotometer equipped with a cooled charge-coupled device camera and excited with a 514.5 nm wavelength solid-state laser source.

### 2.4. Electrochemical Characterization

The electrodes were tested by chrono-amperometry, linear sweep voltammetry and electrochemical impedance spectroscopy. The measurements took place in a 0.1 M KHCO_3_ aqueous solution continuously saturated by CO_2_, with a flux of 20 mL min^−1^. The working electrode was a catalyst-coated carbon paper, while a Pt coil was used as counter electrode and Ag/AgCl (3M NaCl) was used as reference electrode. For all measurements 85% of the series resistance was compensated. Before being tested, the samples were reduced for 20 min at −0.99 V. All the applied potentials reported refer to the reversible hydrogen electrode (RHE).

All measurements except CA were performed in a single cell with a Metrohm multi Autolab/M204 potentiostat/galvanostat, with electrodes geometric area of 0.2 cm^2^. LSV was used to study the electrode catalytic performances toward CO_2_RR in the potential range from 0.5 to −1.2 V at a scan rate of 1 mV s^−1^. EIS was performed with an AC signal of 10 mV of amplitude in 10^−1^–10^4^ Hz frequency range.

CA analysis was conducted with cathodes having 1.5 cm^2^ geometric area in a custom-made two compartment cell, separated by a proton exchange Nafion membrane N 117 (Sigma-Aldrich). The applied potential was provided by a CHI760D electrochemical workstation, while the already described Pt and Ag/AgCl were employed as counter and reference electrodes. Gas phase products were measured online using a micro gas chromatograph (μGC, Fusion, INFICON, Bad Ragaz, Switzerland). The gas is filtered at the inlet to remove the humidity and then it flows through two channels, equipped with a 10-m Rt-Molsieve 5A column and with an 8-m Rt-QBond column, respectively. Both columns are equipped with a microthermal conductivity detectors (micro-TCD). Liquid products were detected using a High-Performance Liquid Chromatograph (Ultimate 3000 HPLC, Thermo Fisher Scientific, Waltham, MA, USA) with a UV-Vis Detector set at 210 nm by using a ReproGel (300 mm × 8 mm) column, with 9.0 mM H_2_SO_4_ (flow rate of 1.0 mL·min^−1^) as mobile phase.

## 3. Results and Discussion

### 3.1. Characterization of the Prepared SnO_2_ Nanocatalysts Doped with Zn and Ti

**Figure 1:**
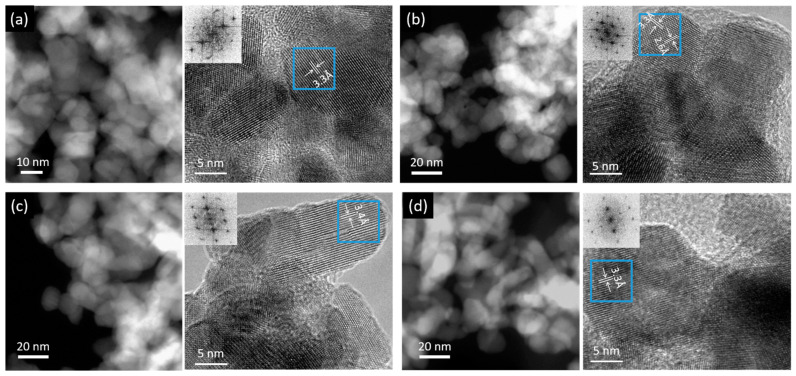
STEM and HRTEM images with corresponding Fast Fourier Transforms (FFT) of all studied catalysts: (**a**) Reference, (**b**) TiWet, (**c**) ZnALD and (**d**) ZnWet. In HRTEM images, interplanar spacings measured from FFT are provided (~3.3 Å (110), ~2.6 Å (101), family of planes of the SnO_2_ structure).

The morphology of the prepared samples can be seen in FESEM images in Figure S1 of the Supporting information (SI) and from TEM characterization in Figure 1. The reference sample (Figure 1a and Figure S1a) shows an irregular porous structure, which is typical of the SnO_x_ prepared by anodic oxidation [12]. The pore walls are made of connected nanocrystals as can be seen from the STEM image Figure 1a. HRTEM image shows nanocrystals of a good crystallinity, with lattice fringes with regular spacing, which are consistent with interplanar distances of SnO_2_ (Tin Oxide, JCPDS 00-041-1445). This is also confirmed by the Fast Fourier Transform (FFT, inset of Figure 1a) of the area in the blue square, which shows well defined spots of SnO_2_ crystal. The Selected Area Electron Diffraction (SAED) pattern (Figure S2) shows well defined diffraction rings, confirming the presence of polycrystalline SnO_2_ with highly crystalline structure.

The electron microscopy characterization of the doped samples (Figure S1b–d and Figure 1b−d) shows that the morphology of the Reference sample is preserved. This was highly desirable as the mesoporous structure, for example, created during the anodic oxidation synthesis, allows easy access of the electrolyte to the catalytic sites and efficient mass diffusion [12,31]. In addition, the electron microscopy images show that there is no evidence of any other structures created. HRTEM images and FFTs show, similarly to the reference sample, that nanocrystals are of a good crystallinity, and lattice fringes with regular spacing are consistent with interplanar distances of SnO_2_. The structural characterization via SAED of the doped samples show the same structure of the reference. There is no detectable evidence of dopant in the diffraction pattern, neither in the form of spots nor rings attributed to metallic dopant or metal-based compounds, nor in the measurable change of the interplanar spacing in comparison to SnO_2_. However, the presence of dopants was confirmed by EDX in the same samples (shown in Figure S3 and discussed below).

**Figure 2:**
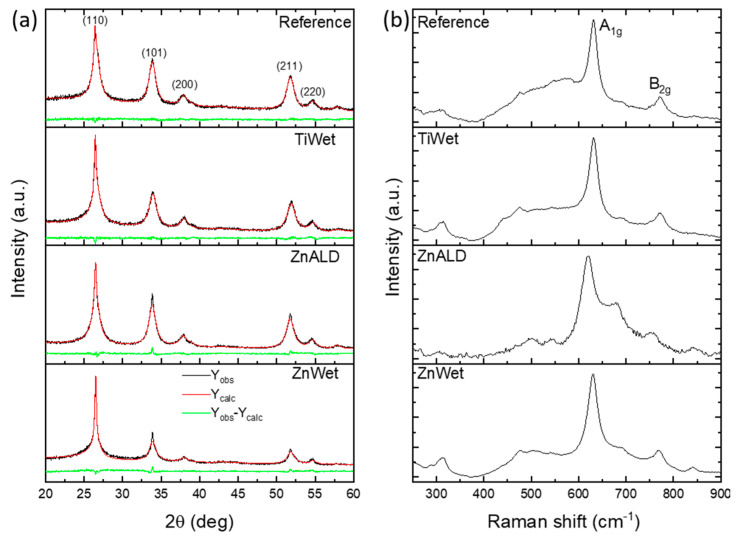
XRD spectra (**a**) and Raman spectra (**b**) obtained from each sample.

The crystalline structure of the studied electrodes was also investigated by XRD. Rietveld refinement for all samples was utilized to deeply investigate the structural properties of the catalysts. In Figure 2a experimental data (Y_obs_) are shown as a black line, calculated intensities are shown as a red line (Y_calc_), while green line represents the difference between the measured and calculated intensities (Y_obs_-Y_calc_). It is important to note that XRD was performed on the as-prepared electrodes and therefore we observe the contribution of the GDL (with the peaks at 26.6° and 54.7° as shown in Figure S4), on which the catalyst is deposited, with the most intense (110) peak of SnO_2_ being at the same 2θ position as this of GDL. For the reference sample, all the observed diffraction peaks can be indexed to tetragonal rutile SnO_2_ with P42/mnm space group (tin oxide, JCPDS 00-041-1445). The same peaks and therefore the same crystalline structure was identified within the spectra of doped samples. For all catalysts, the peak shape gives evidence of small coherent diffraction domains, consistent with HRTEM analysis. Similarly, as in the TEM and SAED analysis, there is no evidence of dopant in terms of the appearance of diffraction peaks which could be attributed to metallic dopant or metal-based compounds. This indicates that metal is incorporated into the tin oxide crystalline lattice. The fitting quality of the experimental data is good, as Rietveld refinement R-factors have been reduced to minimum. The lattice parameter (a) and the coherent diffraction domain size (D) were calculated for each sample, and the values are given in Table S1. It can be observed that the lattice parameter of the samples shows small variations due to the different ionic radii of Zn^2+^ (0.74 Å), Ti^4+^ (0.61 Å) and Sn^4+^ (0.71 Å). It is slightly reduced for Ti doping, causing the reduction in the unit cell volume. Ran et al. showed a slight increase in 2θ XRD peaks position with an increase in Ti doping content, assigned to a decrease in lattice constants due to the diameter of Ti^4+^ being smaller than that of Sn^4+^ [20]. The difference between Zn^2+^ and Sn^4+^ is relatively smaller, and less variation was observed. Slight change in the unit cell size was observed also by Mishra et al. [32].

Raman spectroscopy demonstrates that the Reference sample shows two principal vibrational modes typical of SnO_2_, positioned at about 630 cm^−1^ (A_1g_ mode) and 770 cm^−1^ (B_2g_ mode) that are associated with symmetric and asymmetric stretching of Sn–O bonds (Figure 2b). This confirms the presence of crystalline tin oxide in tetragonal rutile polymorph in agreement with the XRD results. The introduction of dopant species into the SnO_2_ crystal structure deeply affects the lattice vibrational modes, which may result in an increase of peak linewidth and a shift of peak position (see Figure S5 in SI) (which can be either blue or red shift, depending on the dominant mechanism [33]). These effects can be related to lattice distortion due to substitution of Sn by dopant ions and to an increase of the oxygen vacancies concentration [34]. These phenomena mainly affect the reported Raman spectra with an increasing tendency of peak linewidth, which can be related to the size effect due to crystallite size reduction and/or to the change in the particle size [35] and the strain associated with the segregation of dopant ions at SnO_2_ surfaces [36]. In particular, mechanical strain is expected in Ti-doped samples due to lattice unit cell size variation. In addition, the ZnALD sample, which shows also a consistent shift of peaks, the other doped samples show only a slight redshift of peaks position, which is likely the result of a combination of different mechanisms.

**Figure 3:**
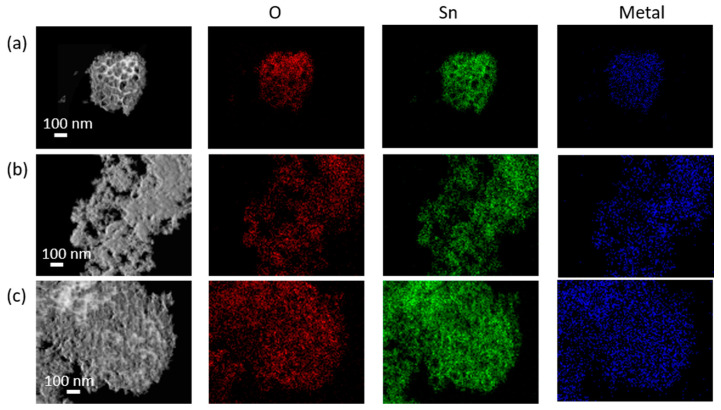
STEM HAADF images and corresponding EDX elemental mapping of doped samples: (**a**) TiWet, (**b**) ZnALD and (**c**) ZnWet.

EDX was performed on all samples to confirm the composition, namely to investigate the presence of the inserted element as shown in Figure S3. The spectrum of the reference sample revealed elemental Sn and O with some C, originating from the sample, and Fe, Co and Cu peaks attributable to the experimental set-up. The investigation of the doped samples confirms the presence of the dopant element within the structure. The relative atomic concentrations for Sn and doping atoms are reported in Table S2, and will be discussed in the later section together with the XPS compositional analysis. Furthermore, scanning transmission electron microscopy high-angle annular dark field (STEM-HAADF) imaging and corresponding elemental mapping were carried out, as shown in Figure 3. These maps confirm the uniform spatial distribution of Zn and Ti in doped SnO_2_ samples.

The surface chemical composition and valence states were further investigated by X-ray photoelectron spectroscopy. Survey spectra (not reported) evidence the presence of C, O, Sn, and doping atoms (Zn and Ti) as reported in Table S3. Higher relative atomic concentration of the dopants was observed by XPS respectively to EDX, due to the different volume investigated by the two techniques. XPS is more sensitive to the first few layers of the materials, thus providing an accurate surface composition while EDX has a larger penetration depth (>1μm at applied conditions). The high concentration of the doping atoms in Zn catalysts and the differences in atomic concentration of the doping atoms, measured by XPS and EDX, suggests a sort of surface segregation phenomenon for Zn, for both synthesis methods (wet and ALD). The amount of Ti is an order of magnitude lower, and is similar for both XPS and EDX, which suggests that Ti is homogeneously dispersed in the sample.

**Figure 4:**
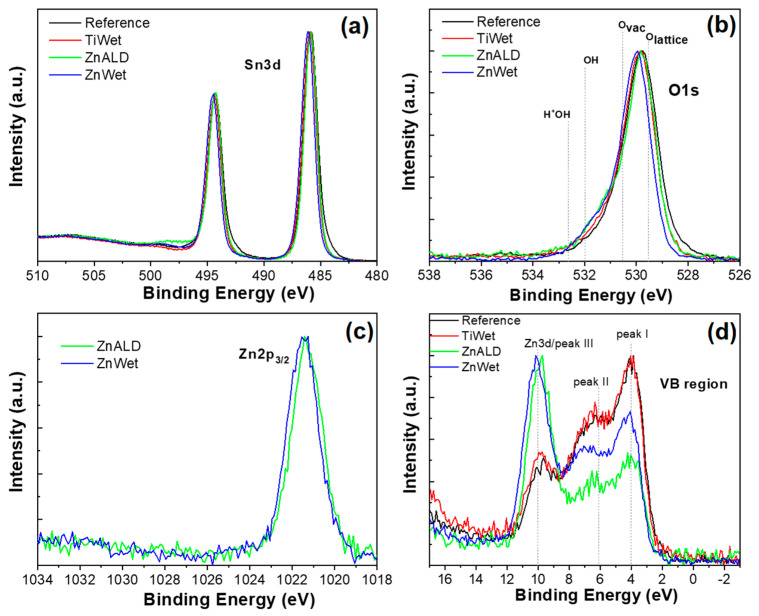
XPS HR spectra for Reference, TiWet, ZnALD and ZnWet samples: (**a**) Sn3d, (**b**) O1s, (**c**) Zn2p3/2, and (**d**) Valence Band (VB) region.

The oxidation state and chemical surrounding of each element detected onto samples surface can be inferred by the analysis of High Resolution (HR) spectra. As reported in Figure 4a, Sn3d doublet shows the typical spectra of tin oxide (Sn3d_5/2_ peak located at 486.0 eV), well overlapped for all doped samples, while the Reference sample shows an asymmetric peak with a small enlargement at lower binding energy, due to the presence of a second oxidation state (peak at 484.2 eV), which can be attributed to Sn(0) [37]. The O1s HR spectra are shown in Figure 4b. The experimental data were fitted using four Voigt functions in order to reproduce the experimental curves. These result in four peaks assigned to: (I) lattice oxide around 529.5 eV; (II) oxygen surface vacancy at 530.5 eV; (III) hydroxide at 532 eV; (IV) protonated OH groups at 532.5 eV, as reported also in Table S4 [38]. Considering only the O atoms related to the lattice and not the adsorbed species, the ratio between peak II and (peak I + peak II) gives an indication about the number of O vacancies (O_vac_) present at the surface, as reported in the last column of Table S4. This parameter shows a clearly increasing trend, starting from the Reference sample that shows the lowest amount of O_vac_, until the ZnWet sample that shows the highest amount of O_vac_, implying an increase of about 2.5 times with respect to the bare sample. For Zn-doped samples (Wet and ALD), also the Zn2p_3/2_ photoelectron peaks have been evaluated (Figure 4c), showing a quite good overlapping at 1021.4 eV peak attributed to Zn^2+^ [39]. Similarly, for TiWet sample, also the Ti2p_3/2_ region has been evaluated (data not reported), showing a single peak due to Ti^4+^ at 458.3 eV [39].

Another valuable analysis of the doping process is the examination of the Valence Band (VB) region by XPS, because VB represents the experimental evidence of the density of states (DOS), which is sensitive to band gap modification. Moreover, the analysis of VB region is useful to distinguish between SnO and SnO_2_ oxides, which show similar chemical shift energies in their photoelectron peaks (Sn3d doublet) but quite different VB spectra shapes [40]. As reported in Figure 4d, the VB region displays the typical SnO_2_ three-peaked structure, although for Zn-doped samples the presence of Zn3d peak around 10 eV masks the peak III of tin oxide. While Reference and TiWet samples present quite well overlapping spectra, Zn-doped samples show a shift toward higher binding energies (Δmax = 0.18 eV). A similar result has been already found in literature by Egdell et al. [41] in their study related to Sb-doped SnO_2_ materials. In that case, the experimental results were explained by the presence of segregated doping atoms at the surface, a phenomenon which induces a shrinkage in the energy gap and a subsequent shift towards higher binding energies of the VB onset.

### 3.2. Electrochemical Behavior for CO_2_ Electroreduction

The electrode performance towards the CO_2_RR was firstly studied using LSV at 1 mV s^−1^ in electrolyte saturated with CO_2_ or N_2_ as shown in Figure S6 in SI. For all tested electrodes, the current starts to grow at a relatively small potential due to hydrogen evolution reaction (HER) and CO_2_RR onsets. The Reference electrode displays higher geometric current densities in the electrolyte saturated by CO_2_ than in the one saturated by N_2_ in the range between −0.6 V and −1.12 V, while this feature is exhibited by all doped samples for all the applied potentials below −0.65 V vs. RHE. This suggests that all the catalysts are more selective towards CO_2_RR with respect to the HER.

**Figure 5:**
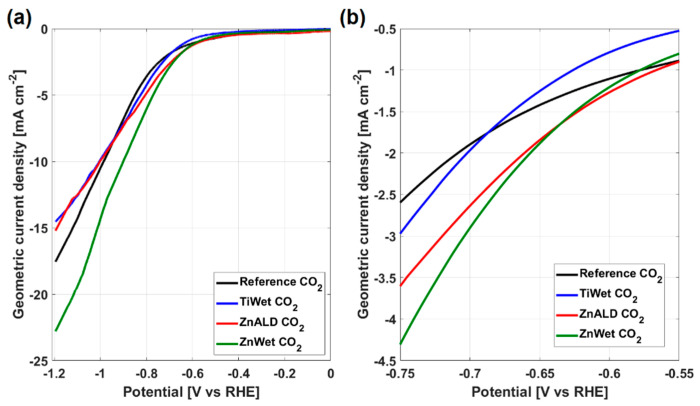
LSV in CO_2_ saturated electrolyte for all electrodes (**a**) in the full potential range (−1.2, 0) V, (**b**) zoom in the potential range of (−0.75, −0.55) V.

To discuss the electrodes’ electrochemical responses, their LSV in CO_2_ are reported and compared in Figure 5a.

It is evident that doped samples have higher geometric current density with respect to the Reference sample and the ZnWet shows the highest values among all the electrodes. The insertion of dopants increases the current in a potential range close to the onset potential, as highlighted in Figure 5b. LSV shows that Zn-doped samples have an improved activity for CO_2_RR with respect to Reference electrode, which is in line with theoretical predictions [13]. This proves that doping SnO_2_ with certain elements results in a shift of the onset potential, which in turn results from a change in the binding energy of the intermediates on the modified surface (as discussed below). We observed that the same current is reached at different potential values for different electrodes, with lowest values of overpotential for the doped samples. This indicates that the same activity is obtained at less negative potentials. For example, the current of 2 mA cm^−2^ is achieved at potential of −0.66 V in ZnWet electrode, which is higher than −0.72 V for the Reference electrode (the same current is achieved at potentials of −0.70 V and −0.66 V for TiWet and ZnALD, respectively). It can therefore be concluded that the doped SnO_2_ electrodes show higher activity at lower overpotentials.

EIS measurements were carried out in order to separate the different contributions to the total geometric current for all the electrodes. A comparison of the Nyquist plots acquired at −0.59 V is reported in Figure S7a as an example. At all potentials, all the spectra are mainly constituted by a high frequency arc, related to the charge transport inside the catalysts, and a low frequency (incomplete) arc, related to the charge transfer at the catalyst/electrolyte interface. An additional feature is the distance between the origin of axis and the beginning of the high frequency arc, which is associated to the electrolyte conductivity [42]. It is worth to notice that the diffusion of charges cannot be appreciated due to low frequency (0.1 Hz) limitations [12]. The experimental data were fitted employing the equivalent electric circuit shown in the inset of Figure S7a, in order to obtain the parameters related to the different processes, namely the high frequency (*R*_t_ and *C*_t_) and low frequency (*R*_ct_ and *C*_dl_) ones, including the series resistance *R*_s_ [43]. Since all the parameters except from the charge transfer resistance R_ct_ exhibit negligible dependence on the applied potential [44], the values related to the measurement acquired at −0.59 V are reported as examples in Table S5. On the other hand, of particular interest is the charge transfer resistance *R*_ct_, whose values for all the electrodes are shown in Figure S7b as a function of the potential. The typical exponential behavior can be appreciated in the semi-log plots, with values that decrease while reducing the potential. Moreover, all the doped samples are characterized by lower *R*_ct_ with respect to the reference electrode, implying that the insertion of dopant atoms is effective in improving the activity of SnO_2_. It is also important to highlight that another beneficial effect of the doping process is to increase the electrode conductivity, as witnessed by the reduced *R*_t_ values with respect to reference sample (see Table S5).

Electrochemical active surface area (ECSA) is of high importance in the investigation and development of catalysts. In the studied electrodes, the complexity of the composition does not permit the exact estimation of this parameter. However, as the electrodes are prepared in the same manner and the only difference is the catalyst itself, a comparison between the different electrodes is anyway possible through the double layer capacitance *C*_dl_. It is worth to notice that, beside cyclic voltammetry measurements carried out in non-Faradaic region, ECSA values can be obtained also exploiting the EIS technique [45]. In this framework, electrochemical impedance spectroscopy evaluations show that the *C*_dl_ of electrodes containing doped SnO_2_ is higher compared to the Reference. The electrode with the zinc insertion has a value of *C*_dl_ almost twice (e.g., 20.6 mF cm^−2^ for ZnWet) as compared to the Reference (12.3 mF cm^−2^). This implies that the modified catalysts have an improvement in the ECSA, which is also directly related to an increase of the current of ZnWet sample in CO_2_. The tin oxide catalytic centres have been identified in oxygen vacancies and grain boundaries defects [46]. Currently, it is not possible to determine if the insertion of the dopants generates new active sites. However, it increases the oxygen vacancies concentration, as shown by XPS. In any case, the ZnWet electrode shows the best catalytic performance.

**Figure 6:**
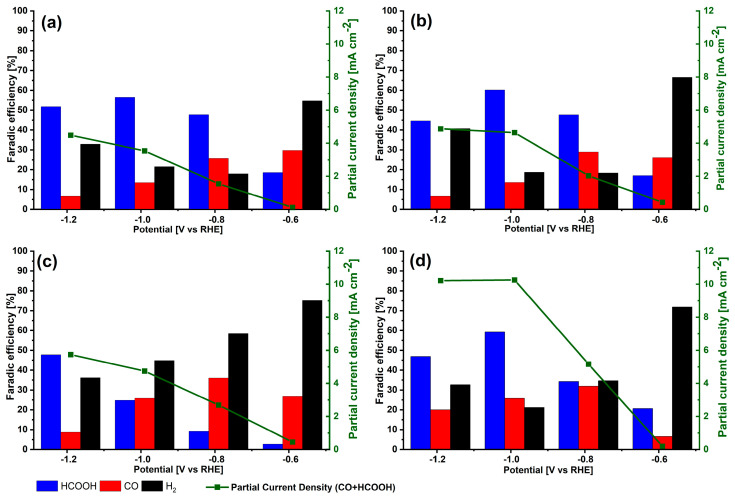
Faradic efficiencies for CO, HCOOH and H_2_ formation and partial current density for electrodes: (**a**) Reference, (**b**) TiWet, (**c**) ZnALD and (**d**) ZnWet.

To compare the performance of the electrodes toward CO_2_RR, CA measurements were carried out in CO_2_ saturated electrolyte (Figure S8 in SI). For all tested electrodes, the current density is low at −0.59 V and it increases with decreasing applied potential. Compared to the Reference electrode, the doped electrodes display a much higher current density at −0.79 V and maintain this advantage at more negative potentials. The highest currents were observed for ZnWet electrode, which is consistent with LSV curves. A measurement of the produced H_2_, CO and HCOOH was determined by μGC and HPLC. The faradaic efficiencies together with partial current densities for CO_2_RR products are shown in Figure 6.

The Reference electrode shows the expected trend: by decreasing the potential, CO_2_RR overcame the HER [12]. The FE value for H_2_ evolution is above 50% at −0.59 V, and it reduces to about 20% and increases again to value of 33% at −1.19 V. The FE for CO production decreases from 30% to 7% in the tested potential range. The CO_2_RR products (CO + HCOOH) reach their highest FE of about 70% at −0.79 V and −0.99 V.

The TiWet electrode shows similar behaviour with respect to the Reference electrode. At the potential of −0.59 V, HER is dominant and the best catalytic properties are obtained at lower potentials. At −0.79 V the TiWet shows the highest FE related to CO_2_RR (77%); however, the partial current density towards CO_2_RR is relatively small. By decreasing the applied potential, the production of HCOOH becomes dominant. TiWet electrode shows FEs similar to those of Reference in all tested potential range, however an improvement can be seen in partial current density for CO_2_RR, which is highest at −0.79 V and −0.99 V.

ZnALD shows quite high FE for H_2_ in all potential range, and it dominates at both -0.59 V and −0.79 V, which is dissimilar to the Reference and TiWet, where it dominated only at −0.59 V. The FE for CO is higher than that of the Reference and TiWet, with the highest value of 36% at −0.79 V. However, the HCOOH production is very limited at all potentials besides −1.19 V, where it reaches 48%.

In ZnWet electrode, similarly to others, at the potential of −0.59 V HER is dominant and the best catalytic properties are obtained at lower potentials. At all potentials besides −0.59 V the FE for CO is higher than that of the Reference and TiWet, and similar to that of ZnALD. This could be related to a change in the binding energy for intermediates in Zn-doped SnO_2_ [46], which influences the reaction path, as discussed below in the reaction mechanism overview. Overall, at −0.99 V, ZnWet has the highest FE of around 80% towards CO_2_RR products, about 10% higher than the Reference sample. The partial current density for CO_2_RR at this potential is 10.3 mA cm^−2^, which is almost triple with respect to 3.5 mA cm^−2^ of the Reference.

In addition, we performed a stability test on this sample, reported in Figure S9, the sample maintained a quite stable current density of about 11.7 mA cm^−2^ and retained constant faradaic efficiencies toward CO (19%) and formic acid (61%), in agreement with the results shown above in Figure 6.

**Figure 7:**
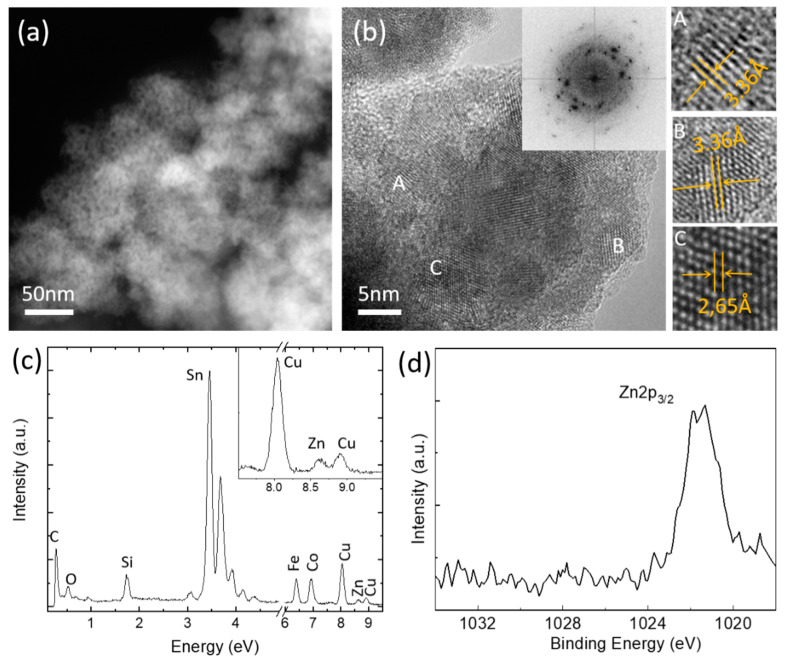
Characterization of tested ZnWet catalyst: (**a**) STEM image, (**b**) HRTEM images with corresponding FFT. Higher magnification images of A, B and C particles are also shown, and in these interplanar spacings calculated from FFT are provided (~3.3 Å (110), ~2.6 Å (101) family of planes of the SnO_2_ structure); (**c**) EDX and (**d**) XPS.

The morphology, phase changes, and crystallinity of the tested catalysts were studied via electron microscopy, EDX and XPS. Morphological characterization, shown in Figure 7 and Figure S10 in SI, evidences that the SnO_2_ catalyst and doped SnO_2_ catalysts have changed after CO_2_ reduction reaction. On the basis of the HRTEM and STEM measurements, we observed that the size of the original particles decreases while the catalytic activity proceeds, and they tend to aggregate in bigger particles. The HRTEM of all catalysts after electrochemical testing evidences that the small particles are crystalline. This is confirmed by the FFT, in the insets of the HRTEM images, showing the ring pattern and confirming the random crystallites orientation. These were indexed with the lattice parameters of SnO_2_.

Moreover, the presence of the doping elements in the catalysts after CO_2_RR was investigated by EDX spectroscopy, (the representative spectra are provided in Figure 7c for ZnWet catalyst and Figure S11 in SI for the other catalysts) and XPS (Figure 7d). EDX and XPS confirm the presence of the dopant within the structure (Tables S6 and S7, respectively); however, the quantity is lower than in the pristine catalyst. The dopant leaching is a known issue in various systems during electrochemical reaction [47,48]. We believe that under the reduction potential, the material changes and part of the dopant gets lost, which influences long term stability and therefore this should be wider investigated in order to further improve the performances of the doped catalysts.

In order to optimize the catalyst design, it is essential to understand the CO_2_RR to HCOOH and CO reaction mechanism, which was recently deeply studied. The in situ/in operando studies helped resolve the uncertainty in regards to whether the reduced or metastable surface is active for CO_2_RR to HCOOH and CO. These studies give a possibility of simultaneous characterizations of the catalysts under actual reaction conditions, which in turn gives the possibility to identify the active sites and elucidate the reaction mechanisms by knowing reaction intermediates under reactive conditions [49]. The in situ studies by Raman spectroscopy [50] and ATR−IR spectroscopy [51] show that at potentials lower than −0.79 V vs. RHE the reduction of SnO_x_ to Sn happens, and a decreased FE for formate production is observed. These studies prove that the CO_2_RR to HCOOH and CO happens at the metastable surface, and several reaction pathways have been identified for tin oxide [46,52,53]. The formation of HCOOH and CO proceeds through possible OCHO* and COOH* intermediates and depends on the initial binding mode of the first intermediate of CO_2_ reduction. Among those, the most stable is OCHO*: it produces formic acid after the second proton and electron transfer, and it is favoured at lower potentials [46]. COOH* might create CO and formic acid depending on the bonding strength [46,52,53,54]. A huge difference on intermediates, and therefore on selectivity and activity, is strictly related to the surface oxidation state. Li et al., in the study on the effect of tuning the tin oxide’ oxygen vacancies on the CO_2_RR, showed that the oxygen vacancies regulate the catalyst selectivity by increasing the intermediates adsorption strength, and this boosts the formic acid production. This effect is valid until a maximum value is reached, and then it vanishes [54]. The active sites play a key role in the reaction, and the hydroxide groups are dominant at high potentials, but below −0.6 V the oxygen vacancies become dominant [46]. The increased number of oxygen vacancies in the doped SnO_2_, as confirmed by XPS measurements, is believed to contribute to the increased activity of the doped catalysts both from the theoretical studies as specified above [54] and experimental works [14]. Wei et al. showed improved performance in Mn-doped SnO_2_, ascribed to the increased number of oxygen vacancies, suggesting that more unsaturated coordination results in the possibility to adsorb more CO_2_ during the electrolytic process, leading to a more effective conversion process [14]. In this work, however, it is not discussed whether the oxygen vacancies are maintained under reaction conditions or are reduced in the number as suggested by Li et al. [54]. Z. Gu et al. studied CuO_x_ for CO_2_ reduction to C_2_ products and show that FE strongly depends on the oxygen vacancy density in CuO_x_, and the worsening of the productivity was associated with the decreased number of oxygen vacancies [55]. This aspect should be investigated in more detail, and possibly the quick progress in situ microscopy and spectroscopy techniques will help to shed some light on this aspect and lead to optimization of the catalysts design. As of today, there are no in operando studies on doped SnO_2_, which would help to understand the reduction mechanism. However, we suppose that doping results in the different intermediates binding energies in the studied catalysts [56]. It was actually shown by Saravanan et al. that the modifications of the SnO_2_ catalyst (for example the insertion of the dopant atoms) decrease the intermediates bonding energy with the catalysts surface [13], resulting in the reduction of the overpotential necessary for the CO_2_RR. Moreover, the adsorption energies of H*, OCHO*, and COOH* strictly influence the substrate CO_2_RR selectivity [56]. For this reason, the addition of Zn and Ti, which reduces the bonding energies of the intermediates, is also believed to increase the product selectivity towards CO [56], which is consistent with the experimental results observed for Zn-doped SnO_2_.

**Figure 8:**
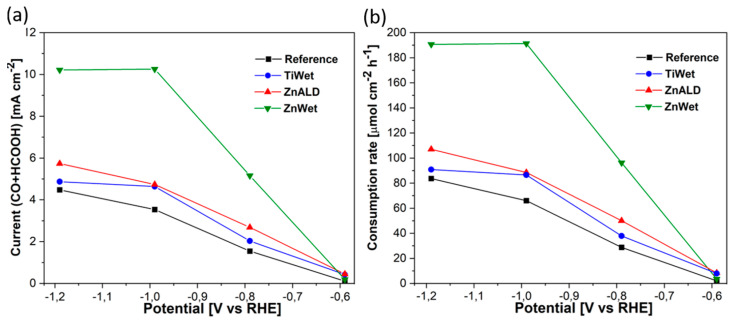
(**a**) CO_2_RR partial current density and (**b**) CO_2_ consumption rate for all studied electrodes.

Even though all the electrodes demonstrate relatively similar selectivity towards CO_2_RR, the insertion of metal atoms increases the average current density related to CO_2_RR, as evidenced in Figure 8a, and hence the production rates of CO_2_RR products, as shown in Figure S12 in SI. The ZnWet electrode can reach high HCOOH and CO production rate of 191 μmol h^−1^ cm^−2^ at −0.99 V, which is triple with respect to the Reference electrode, showing a production rate of 63 μmol h^−1^cm^−2^ at the same applied potential. The doped materials presented herein demonstrate an enhanced production rate for CO_2_RR compared to the Reference electrode and with reported similar materials [57,58].

A remarkable partial current increase was observed in ZnWet sample, which shows the highest partial current densities towards CO_2_RR at all applied potentials below −0.59 V, that results at least two times higher than that of the Reference electrode. For potential equal or lower than −0.99 V, the partial current density achieves a quasi-plateau of 10.3 mA cm^−2^, suggesting that this electrode suffers from mass diffusion limitations. This is confirmed by CO_2_ consumption rate that has been calculated through the formula [59]:

$$r_{CO_{2}}=\frac{J_{tot}}{2F}\left(FE_{CO}+FE_{HCOOH}\right),$$

where *J_tot_* is the total current density and *F* is the Faraday constant, and is shown in Figure 8b. Thus, even if the total current increases at −1.19 V (Figure S8c in the SI), the current related to CO_2_RR is limited due to the depletion of CO_2_ at active sites. Therefore, the activity and selectivity for the CO_2_RR are reduced and HER increases. A similar behaviour was observed and discussed in our previous works [12,59,60]. The limitation of the production rate of CO and HCOOH related to the mass transport limitation could be overcome by an increase of the CO_2_ availability. This is one of the numerous routes proposed in literature for the optimization of the mass transport, including optimization of the morphology of the catalyst or electrodes, composition of the electrodes and control over local pH. In practice, this can be achieved by an increase of the CO_2_ solubility and employment of high-pressure reactors or three-phase-boundary electrodes and reactors, in which gaseous CO_2_ is directly transported to the catalyst surface through GDL [61]. It was shown by Deng et al. that this change can result in an improvement in a Bi_2_O_3_@C−800 catalyst performance from FEHCOOH of 92% with a partial current density of 7.5 mA cm^−2^ at −0.9 V vs. RHE in the H-type cell, to FEHCOOH of 93% with a high current density of over 150 mA cm^−2^ at the same potential in the flow cell configuration [49].

## 4. Conclusions

Zn and Ti-doped SnO_2_ catalysts have been prepared with simple routes. All doped samples show unaltered morphology with respect to the un-doped SnO_2_. The un-doped SnO_2_ electrode exhibits good selectivity for the CO_2_RR with faradaic efficiencies for HCOOH and CO of 14% and 57%, respectively, and partial current density for CO_2_RR of 3.5 mA cm^−2^ at −0.99 V. The doped electrodes show improved current densities related to CO_2_RR products, up to three times higher than the un-doped sample. The highest current density for CO_2_RR products at a potential of −0.99 V was observed for the ZnWet electrode, with a value of 10.3 mA cm^−2^. Overall, this electrode shows much higher activity than the Reference electrode in all the investigated potential range. The improved performance of the doped SnO_2_ electrodes is attributed to the increased quantity of the oxygen vacancies, which was estimated to be 2.5 times larger in the ZnWet with respect to Reference catalyst.

The present work highlights the importance of rational design of oxide materials for CO_2_RR applications. Catalysts with desired characteristics can be obtained by doping, in order to enhance their efficiency. The proposed doped catalysts are inexpensive and resulted from large scale fabrication method, thus enabling their mass-scale production, opening routes for the real applications in CO_2_ conversion.

## Data Availability

Data is contained within the article or supplementary material.

## Supplementary Materials

The following are available online at https://www.mdpi.com/article/10.3390/ma14092354/s1, Figure S1: FESEM images of all studied catalysts. Figure S2: Selected Area Electron Diffraction patterns of all studied catalysts. Figure S3: EDX spectra of all studied catalysts. Figure S4: XRD spectrum of the GDL substrate. Table S1: Rietveld analysis. Figure S5: Analysis of Raman peak parameters. Table S2: EDX relative atomic concentration. Table S3: XPS relative atomic concentration. Table S4: XPS HR O1s spectra deconvolution procedures. Figure S6: LSV in N_2_ and CO_2_ saturated 0.1 M KHCO_3_ electrolyte for all tested electrodes. Figure S7: EIS analysis. Table S5: Electrical parameters obtained from the EIS analysis. Figure S8: CA measurements carried out in CO_2_-saturated electrolyte at different potentials for all studied catalysts. Figure S9: ZnWet stability test. Figure S10: HRTEM images of tested catalysts. Figure S11: EDX spectra of all tested catalysts. Table S6: EDX atomic concentration for reduced electrodes. Table S7: XPS relative atomic concentration for reduced electrodes. Figure S12: CO_2_RR production rate for both HCOOH and CO.
