# Supplementary material for: Zn- and Ti-Doped SnO2 for Enhanced Electroreduction of Carbon Dioxide
Source: Materials (Basel). 2021 May 1;14(9):2354. doi: 10.3390/ma14092354 (PMC8125724; doi:10.3390/ma14092354)
Supplement: Supplementary file 1 [file materials-14-02354-s001.zip › materials-1184195-supplementary.pdf]

materials

MDPI

Supporting Information

Zn- and Ti-Doped SnO2 for Enhanced Electroreduction of Carbon Dioxide

Katarzyna Bejtka [1,*,†], Nicolò B. D. Monti [1,2,†], Adriano Sacco [1], Micaela Castellino [2], Samuele Porro [2], M. Amin Farkhondehfal [1], Juqin Zeng [1], Candido F. Pirri [1,2] and Angelica Chiodoni [1]

[1] Center for Sustainable Future Technologies @POLITO, Istituto Italiano di Tecnologia, Via Livorno 60, 10144 Turin, Italy; nicolo.monti@iit.it (N.B.D.M.); adriano.sacco@iit.it (A.S.); Amin.Farkhondehfal@iit.it (M.A.F.); juqin.zeng@iit.it (J.Z.); fabrizio.pirri@iit.it (C.F.P.); angelica.chiodoni@iit.it (A.C.)

[2] Department of Applied Science and Technology, Politecnico di Torino, C.so Duca degli Abruzzi 24, 10129 Turin, Italy; micaela.castellino@polito.it (M.C.); samuele.porro@polito.it (S.P.)

* Correspondence: katarzyna.bejtka@iit.it

† These authors contributed equally

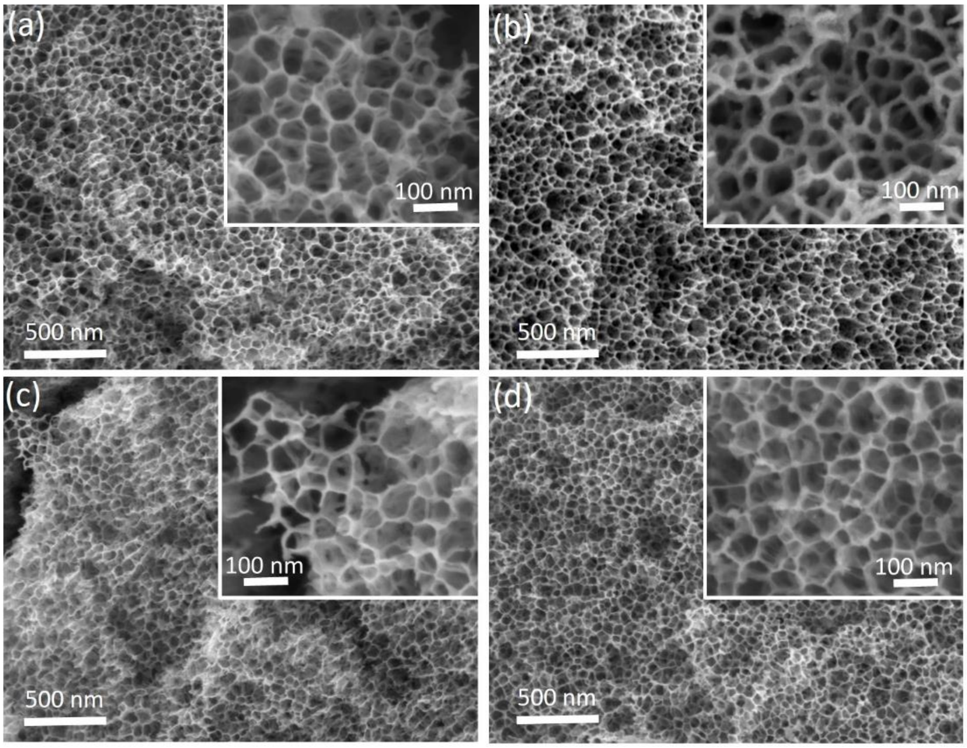

Figure S1. FESEM images of all studied catalysts: (**a**) Reference, (**b**) TiWet, (**c**) ZnALD, and (**d**) ZnWet. Higher magnification images are shown in the insets.

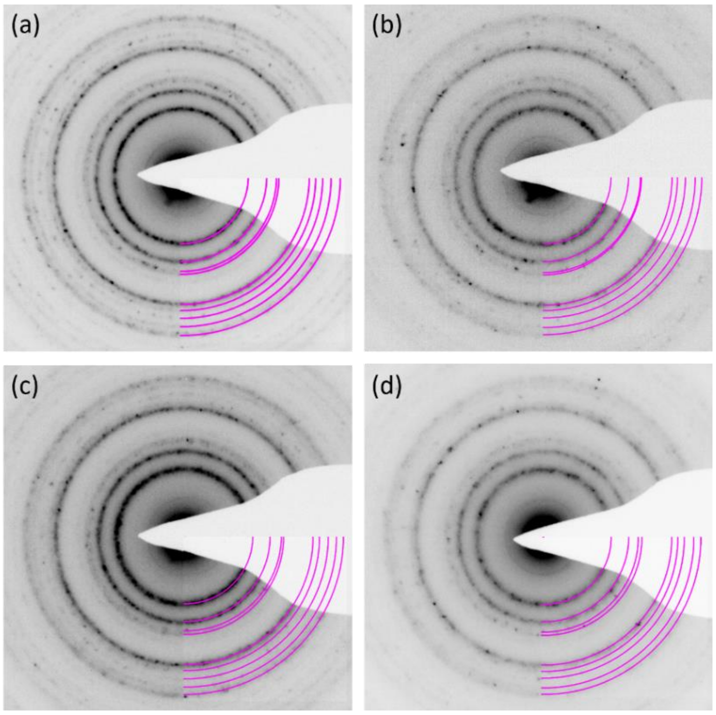

Figure S2. Selected Area Electron Diffraction (SAED) patterns of all studied catalysts: (**a**) Reference, (**b**) TiWet, (**c**) ZnALD and (**d**) ZnWet. The rings in the images were obtained by Circular Hough transform diffraction analysis (A software tool for automated measurement of selected area electron diffraction patterns within Digital Micrograph [1]), and are superimposed on the SAED pattern, showing the position and size of the rings. These are polycrystalline SnO_2 (Tin Oxide, JCPDS 00-041-1445).

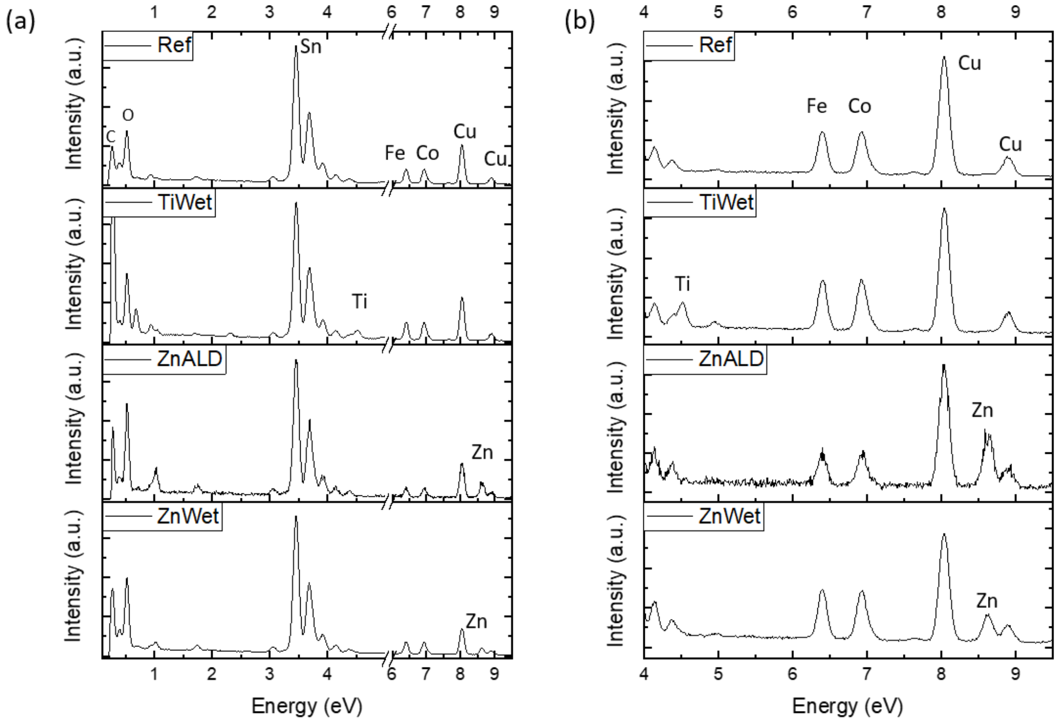

Figure S3. EDX spectrum obtained from a large region of each sample. Fe, Co, and Cu are attributable to experimental setup.

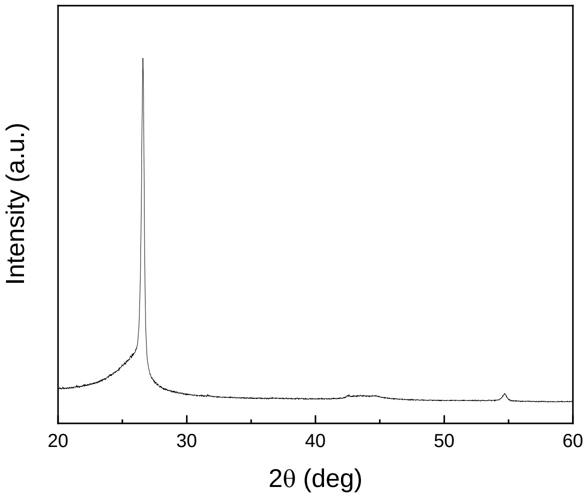

Figure S4. XRD spectrum of the GDL substrate.

Table S1. Rietveld refined structural parameters[a] and the agreement factors (R-factors).

	Reference	TiWet	ZnALD	ZnWet
a=b (Å)	4.740(1)	4.732(1)	4.745(1)	4.734(1)
c (Å)	3.187(1)	3.183(1)	3.186(1)	3.185(1)
Volume cell (Å^3)	71.61	71.25	71.73	71.37
D - crystal size [nm]	10.35(1)	9.23(1)	9.99(1)	9.8(1)
^{2}	1.47	1.51	2.26	2.79
R_{wp} (%)	6.16	6.60	8.72	8.03
R_b (%)	4.75	5.12	6.71	6.47
R_{exp} (%)	5.09	5.37	5.80	4.80

[a] The errors for refined parameters are given in the brackets.

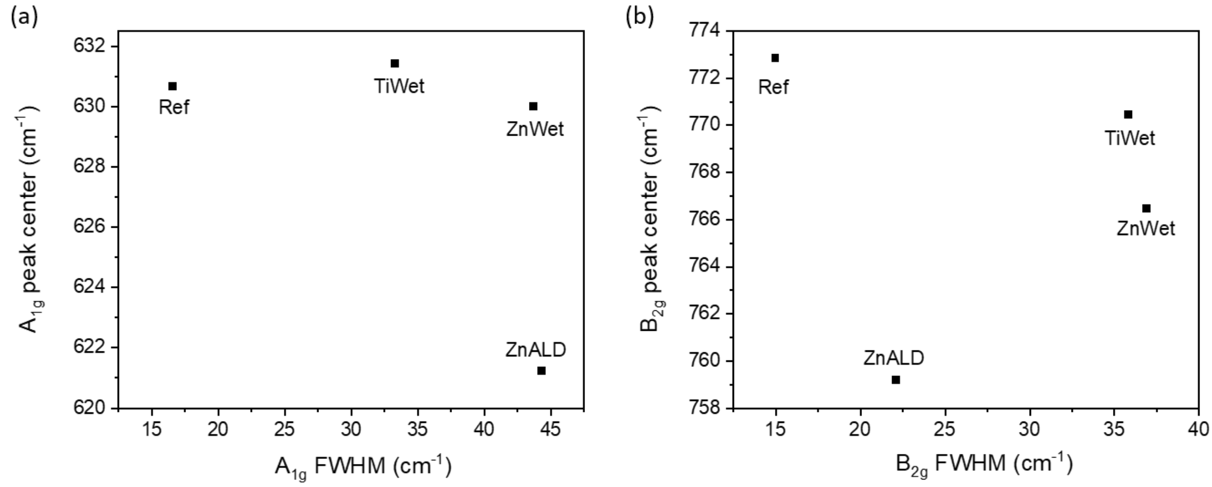

Figure S5. Analysis of Raman peak parameters. (**a**) Peak position of A_{1g} peak versus full width at half maximum of this peak (FWHM), (**b**) peak position of B_{2g} peak versus FWHM of this peak.

Considering the doped samples, Raman data can be used to help understanding whether the dopant entered into the structure or the presence of impurities [2]. The doped

materials show the presence of SnO_2 features in similar position and shape as the Reference sample, without evidence of neither ZnO nor TiO_2 crystals. In order to determine the differences between Reference and doped samples, the reported vibrational modes were curve-fitted to obtain information about peak linewidth (as full width at half maximum, FWHM) and peak center shift. Figure S5 in SI reports two plots showing the peak center position and FWHM of the Reference and doped samples, considering the A_{1g} (Figure S5a in SI) and B_{2g} (Figure S5b in SI) vibrational modes of SnO_2.

Table S2. EDX atomic concentration (at.%) for TiWet, ZnALD, and ZnWet samples.

Sample	Sn (at. %)	Doping (at. %)	Doping/Sn
TiWet	24.6	0.5	0.02
ZnALD	20.7	3.0	0.14
ZnWet	20.7	1.6	0.08

Table S3. XPS relative atomic concentration (at.%) for Reference, TiWet, ZnALD, and ZnWet samples.

Sample	Relative atomic concentration (at.%)				Doping/Sn
	C1s	O1s	Sn3d	Doping atoms	
Reference	19.4	52.1	24.7	-	-
TiWet	20.2	57.3	22.1	Ti: 0.4	0.02
ZnALD	20.8	52.6	20.6	Zn: 6.0	0.29
ZnWet	22.2	51.0	22.8	Zn: 4.0	0.17

Table S4. XPS HR O1s spectra deconvolution procedures and results.

Sample	O1s chemical shift from HR deconvolution [%]				
	Peak 1	Peak II	Peak III	Peak IV	Peak II/ (Peak I+II)
Reference	90.3	6.5	3.2	0	0.07
TiWet	81.0	8.9	6.0	4.1	0.10
ZnALD	76.4	11.7	10.2	1.7	0.13
ZnWet	79.0	14.8	4.5	1.7	0.19
	lattice oxide	oxygen vacancy	hydroxide	protonated OH	

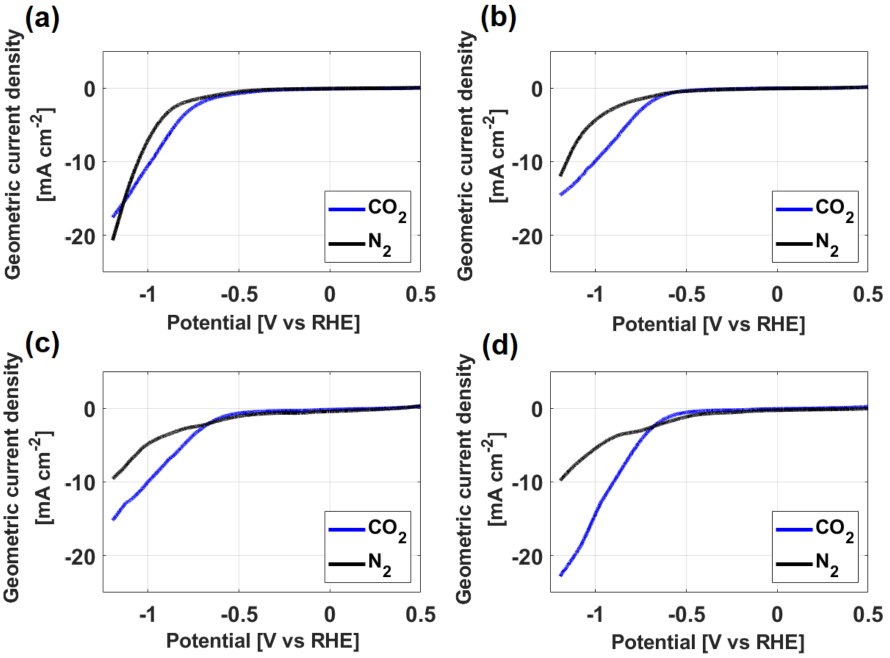

Figure S6. LSV in N_2 and CO_2 saturated 0.1 M $KHCO_3$ electrolyte at a scan rate of 1mV s^{-1}: (**a**) Reference, (**b**) TiWet, (**c**) ZnALD, and (**d**) ZnWet.

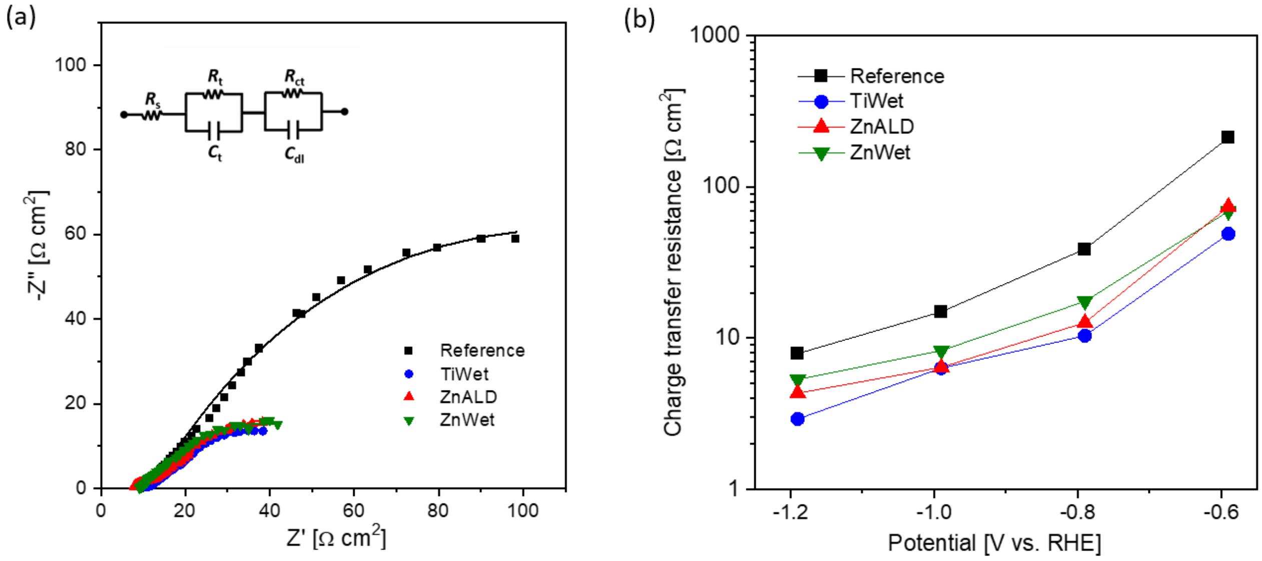

Figure S7. EIS analysis: (**a**) Nyquist plots of the impedance data acquired on the various electrodes in the CO_2-saturated electrolyte at -0.59 V (the points are experimental data; the lines are calculated using the equivalent circuit shown in the inset); (**b**) charge transfer resistances obtained from the EIS fitting procedure on the various electrodes at different potentials.

Table S5. Electrical parameters obtained from the EIS fitting procedure on the various electrodes at -0.59 V (R_s: series resistance; R_t: charge transport resistance; C_t: charge transport capacitance; C_{dl}: double layer capacitance; χ^2: chi-squared error).

Sample	R_s (Ω cm²)	R_t (Ω cm²)	C_t (mF cm⁻²)	C_{dl} (mF cm⁻²)	χ^2 (·10⁻⁴)
Reference	10.8	5.3	0.7	12.3	6.4
TiWet	10.3	2.2	2.4	14.7	4.4
ZnALD	8.1	2.6	1.4	22.3	7.9
ZnWet	9.9	2.7	2.1	20.6	3.6

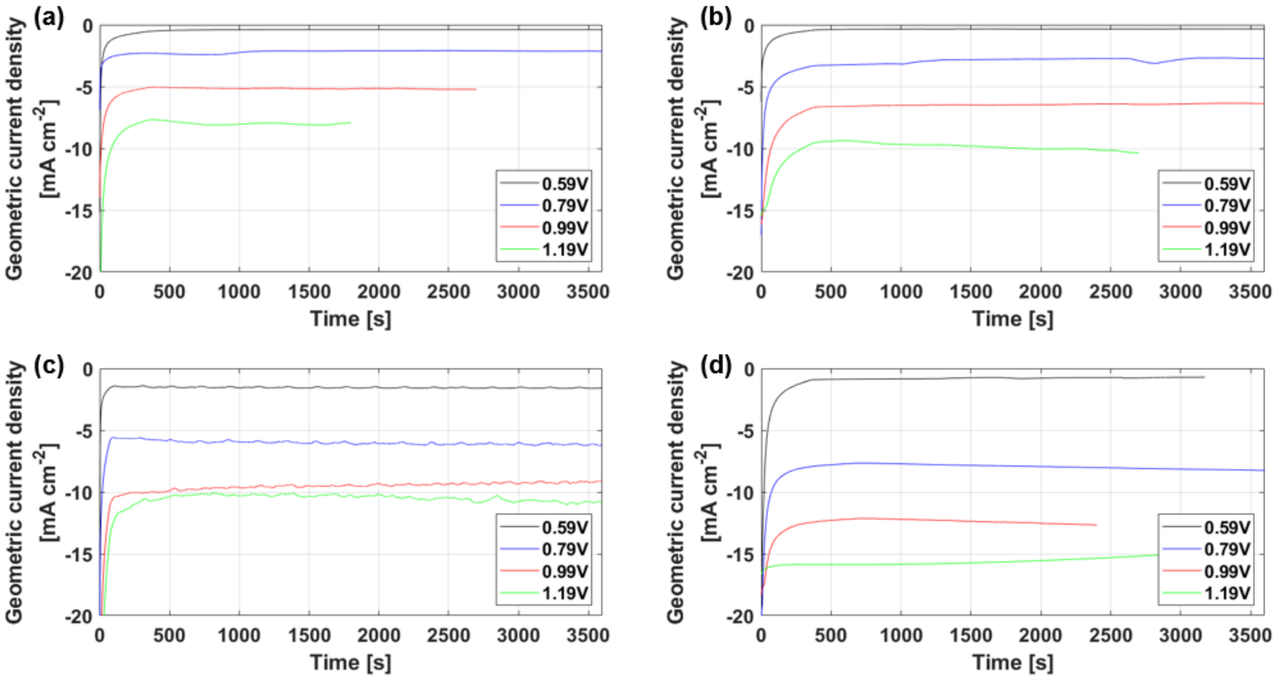

Figure S8. CA measurements carried out in CO_2-saturated 0.1 M $KHCO_3$ aqueous solution at different potentials: (**a**) Reference, (**b**) TiWet, (**c**) ZnALD, and (**d**) ZnWet.

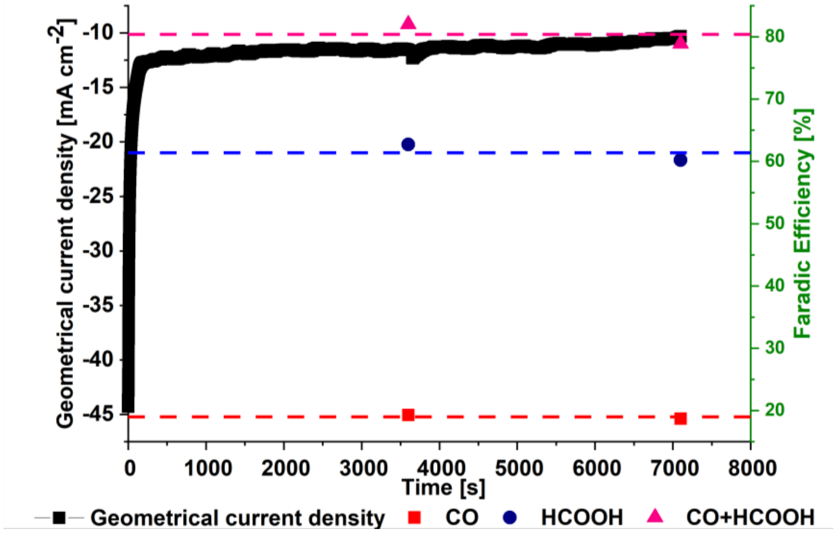

Figure S9. ZnWet stability test and related CO_2RR products faradic efficiency.

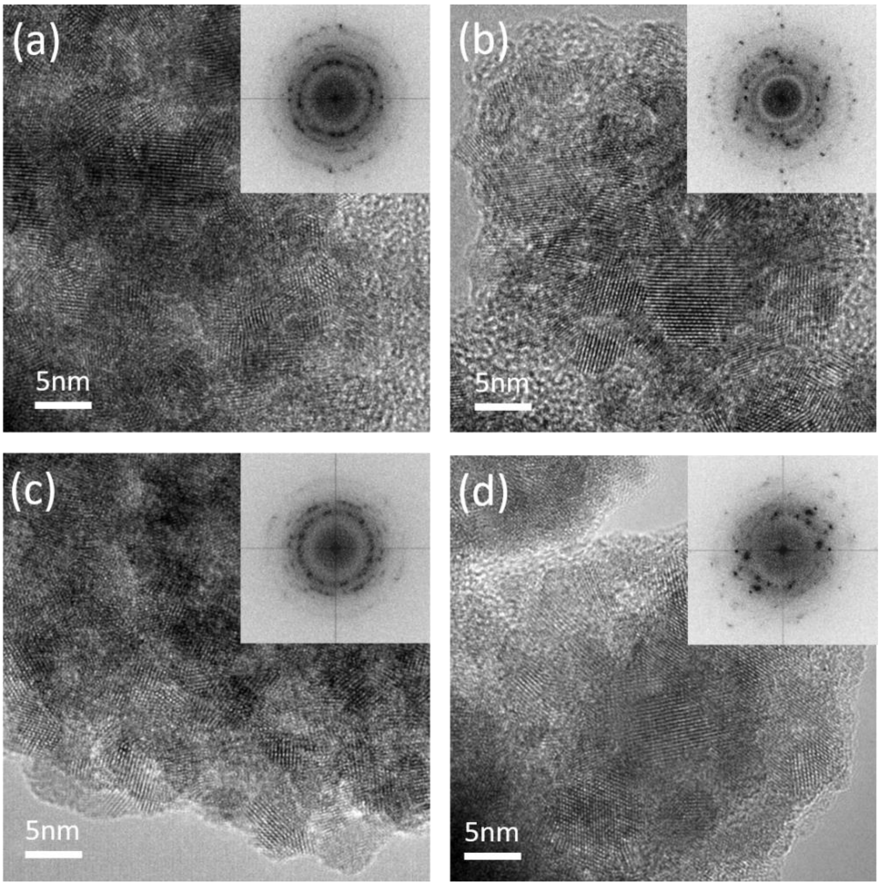

Figure S10. HRTEM images with FFT in the insets of all tested catalysts: (**a**) Reference, (**b**) TiWet, (**c**) ZnALD, and (**d**) ZnWet.

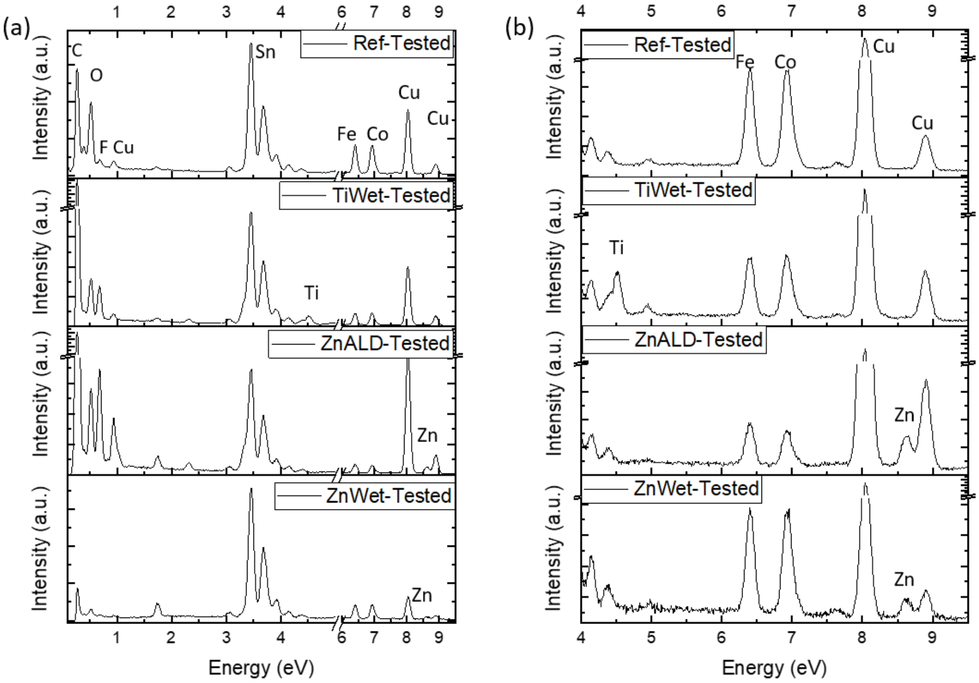

Figure S11. EDX spectrum obtained from a large region of each tested catalyst (**a**) in the full energy range, (**b**) Zoomed to evidence the presence of the dopant elements. Fe, Co, and Cu are attributable to experimental setup.

Table S6. EDX atomic concentration (at.%) for TiWet, ZnALD, and ZnWet tested catalysts.

Sample	Sn (at. %)	Doping (at. %)	Doping/Sn
TiWet	7.3	0.1	0.02
ZnALD	5.7	0.4	0.07
ZnWet	6.3	0.3	0.05

Table S7. XPS relative atomic concentration (at.%) for TiWet, ZnALD, and ZnWet tested catalysts.

Sample	Relative atomic concentration (at.%)				Doping/Sn
	C1s	O1s	Sn3d	Doping atoms	
TiWet	47.6	39.6	12.7	Ti: 0.1	
ZnALD	53.1	38.5	7.9	Zn: 0.5	0.01
ZnWet	70.2	24.9	4.6	Zn: 0.4	0.06

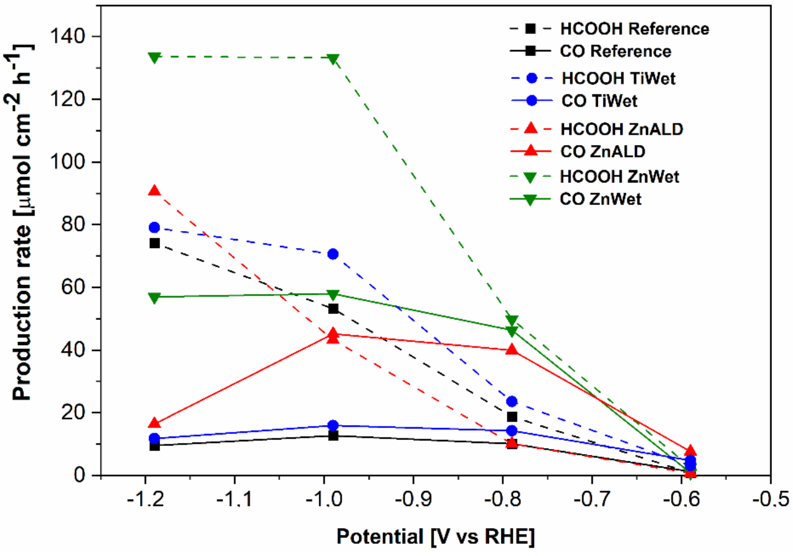

Figure S12. CO_2RR production rate for both HCOOH and CO.

References

1. Mitchell, D.R.G. Circular Hough transform diffraction analysis: A software tool for automated measurement of selected area electron diffraction patterns within Digital Micrograph™. *Ultramicroscopy*, **2008**, 108, 367-374.
2. Aragon, F. H.; Coaquira, J. A. H.; Hidalgo, P.; Da Silva, S. W.; Brito, S. L. M.; Gouvea, D.; Morais, P.C. Evidences of the evolution from solid solution to surface segregation in Ni-doped SnO_2 nanoparticles using Raman spectroscopy. *J. Raman Spectr.* **2010**, 42, 1081-1086.
